# PROTOCOL: Evidence and Gap Map Protocol: Interventions promoting safe water, sanitation, and hygiene for households, communities, schools, and health facilities in low‐ and middle‐income countries

**DOI:** 10.1002/CL2.199

**Published:** 2018-10-08

**Authors:** Hugh Waddington, Hannah Chirgwin, John Eyers, Yashaswini PrasannaKumar, Duae Zehra, Sandy Cairncross

## Background

### The problem

According to the Joint Monitoring Programme (JMP), an estimated 844 million people do not use improved water sources and 2.3 billion lack access to even a basic sanitation service (WHO/UNICEF JMP, 2017). Worldwide, 892 million people still practice open defecation. Rural, poor and, vulnerable households have particularly limited access to adequate facilities and inequities are often regionally focused. Populations in sub‐Saharan Africa and Oceania are lagging behind in access to improved drinking water sources, whilst South Asia and sub‐Saharan Africa have the highest concentrations of open defecation.

Limited, or no, access to safe facilities for eliminating human waste, gathering clean drinking water, or practicing hygienic washing and food preparation practices exposes individuals to higher‐levels of contagious pathogens. There is evidence to suggest that poor water, sanitation, and hygiene (WASH) conditions are associated with high levels of diarrhoeal disease (Clasen et al., 2015; De Buck et al., 2017), respiratory infections (Aiello et al., 2008), parasitic worm (e.g. helminth and schistosome) infections (Ziegelbauer et al., 2012), trachoma (Rabiu et al. 2012), and possibly even tropical enteropathy (Cumming and Cairncross, 2016). Chronic high infection rates are among the leading causes of undernutrition and death in children (Cairncross et al. 2014). Diarrhoeal diseases, in particular, are the second most common cause of death for children under the age of five; diarrhoeal diseases, in particular, are estimated to kill 480,000 children a year (UNICEF, 2018). Beyond the health consequences, poor quality WASH conditions may also lead to long‐term adverse social and economic outcomes including diminished educational attainment (Hennegan et al., 2016), due to both children's school enrolment and attendance as well as teacher attendance, and implications for employment, life‐time wage earnings and income (Hutton et al., 2007; Turley et al. 2013).

Inadequate access affects disadvantaged groups disproportionately, but women and girls are particularly badly affected by the costs of having limited access to WASH facilities. They often carry the majority of the burden associated with collecting water (including time, calories spent, musculoskeletal injuries, risks of assault and attack by humans and wild animals, and road casualties), and can be placed in high‐risk situations when using unsafe places to defecate (Cairncross and Valdmanis, 2006; Cairncross et al., 2010; Sorenson et al., 2011; Sahoo et al., 2015). Women and adolescent girls also experience particular hardships where inadequate WASH facilities constrain menstrual hygiene management (Hennegan et al., 2016; Sumpter and Torondel 2013). There may also be adverse maternal and child health implications due to inadequate WASH services in health facilities and other places of newborn delivery (Benova et al., 2014).

In 2015, more than 150 world leaders adopted the new 2030 Agenda for Sustainable Development, which sets new goals for 2030 that build upon, and go even further, than the Millennium Development Goals (MDGs). Sustainable Development Goal (SDG) 6 aims to ‘ensure the availability and sustainable management of water and sanitation for all’ by 2030 (UN Water, 2018). In order to help achieve these universal targets, which includes reaching the most disadvantaged populations, decision makers need access to high quality evidence on what works in WASH promotion in different contexts, and for different groups of people. Both impact evaluations and evidence syntheses can be useful to decision makers. Single impact studies are useful for providing information on how a programme functions in a specific context; for example, the recent WASH‐Benefits trials were unable to detect effects of combined or single water, sanitation, or hygiene interventions on child linear growth in Bangladesh and Kenya (Luby et al., 2018; Null et al., 2018). However, there has been criticism of the generalisability of the studies and the interventions provided (Cumming and Curtis, 2018; Coffey and Spears, 2018). On the other hand, high quality systematic reviews critically appraise and corroborate the findings from individual studies, as well as providing a steer to decision makers about which findings are generalisable and which are more context‐specific (Waddington et al., 2012).

For policymakers, practitioners and commissioners of research to make informed decisions, they need to be able to identify where high quality evidence exists in usable formats, and where more evidence is needed. There are also concerns about approaches used to measure outcomes in WASH sector primary research, such as self‐ and carer‐reporting of diarrhoeal disease (e.g. Schmidt and Cairncross, 2009). These concerns necessitate, firstly, examining the critically appraised evidence (from systematic reviews) and, secondly, evidence on a wide range of behavioural, health and socio‐economic outcomes.

What remains an issue, therefore, is the extent of evidence on the effectiveness of interventions to improve access to WASH services for households, communities, schools and health facilities on outcomes in the round, and an assessment of what primary and synthesised evidence is still needed across different low‐ and middle‐income countries and regions.

### Scope of the evidence and gap map

Water, sanitation and hygiene (WASH) interventions have two important components to them – the ‘what’ and the ‘how’. The ‘what’ describes the technology that the participants end up with (for example, a latrine) and the ‘how’ describes the mechanism of the intervention (for example, whether toilets are provided on a subsidised basis or at full cost with some form of social marketing). Prior to the early‐2000s, the focus of the conversation was principally on ‘what’ works; research was centred on understanding and demonstrating the short and long term consequences of providing a technology. However, over the last 15 years the conversation has increasingly switched from not just what technology to provide but what is the best way to both get it into the community and have it be regularly used. This has seen the rise of behaviour change and systems‐based approaches. Due to this changing focus, the principal interventions will be defined by the mechanisms (the ‘how’); this means that the evidence gap map will present intervention mechanisms against outcomes. There will then be a filter for the technology provided by the intervention; this will allow for easy comparison of the evidence for different mechanisms of providing, for example, latrines.

Mechanisms for providing WASH technologies can be classified into four main groups; direct provision, health messaging, psychosocial ‘triggering’, and systems‐based interventions. The below definitions have been adapted from relevant literature in the field (De Buck et al., 2017 and Poulos et al., 2006):

- Direct provision mechanisms cover all interventions where hardware (such as a latrine or water purifier) is provided for free and has been chosen by an external authority (such as a non‐governmental organization).
- Health messaging, most often focused on sanitation or hygiene, is typically a directive educational approach designed to help individuals, or communities, improve their health through increasing their knowledge and/or skills.
- Psychosocial ‘triggering’ falls into two subcategories of directive and participative approaches. Both subcategories use behavioural factors which have been derived from psychosocial theories (such as emotions, like disgust and the desire to be a good parent, or social pressure) to motivate behaviour change, rather than reason. An example of this approach is community‐led total sanitation (CLTS) where the community is encouraged to discuss how they would like sanitation practices to change, identify problem areas (e.g. ‘walks of shame’), and use social cohesion and pressure to motivate people to construct latrines and stop practicing open defecation (Kar and Chambers, 2008).
- Systems‐based mechanisms try to change people's behaviour by changing the wider system around them. These approaches include pricing reform, improving operator performance, private sector (PS) and small‐scale independent provider (SSIP) participation, and community driven development (CDD).
- The behavioural change communication (BCC) approaches – health messaging and psychosocial ‘triggering’ – are often combined with both direct provision and systems‐based approaches in an attempt to simultaneously overcome the social and financial barriers to accessing appropriate WASH services.

WASH technologies for household and personal consumption can be classified into four main, related, groups: water quantity, water quality, sanitation hardware and sanitation software (hygiene) (Esrey et al., 1991):

- Water quantity technologies provide a water supply or distribution system. Water may be supplied to communities at source, such as through a public standpipe, or at point‐of‐use (POU), such as being piped directly to households.
- Water quality technologies provide the means to protect water from, or treat water to remove, microbial contaminants. Examples of water treatment technologies include filtration, chlorination, flocculation, solar disinfection, boiling, and pasteurising. Water quality improvements are most commonly undertaken in the household, rather than at the source, though this class of interventions also includes treatment at source and provision of containers for safe transportation and storage of water.
- Sanitation technologies provide means to dispose of excreta (such as faeces), through new or improved latrines or connection of existing latrines to the public sewer.
- Hygiene technologies consist of hygienic practices, and facilitators of these such as soap, hand sanitisers, and washing stations. Hygiene practices are most often focused on handwashing but can also include food hygiene, such as proper food storage and washing dishes appropriately, as well as wearing appropriate footwear, or menstrual hygiene management.

A third important dimension to any intervention is how, or where, participants interact with it in terms of both their social and physical environments. Interventions that seem similar can be very different in nature, and their outcomes not necessarily comparable, due to the space they inhabit. An ecological model can be integrated into the types of technology to reflect where a technology is used. The place of use is important in the WASH sector as it affects the convenience to users, and therefore adoption rates, as well as how the intervention disrupts the causal chain of disease transmission. The four main spaces in which WASH technologies are provided are in the home (for use by an individual household only), in the community (to be shared), at a school, and at a health facility.

Multiple mechanisms can be used in one programme; for example, soap could be directly provided with a social marketing campaign on handwashing. Multiple WASH technologies are also often be provided together in programmes where they are combined. A common example is combined water supply and sanitation (WSS) programmes.

The quality of water supply, sanitation and hygiene facilities – that is, the extent to which they are likely to provide potable drinking water or safe removal of excrement from the human environment, or enable hygienic hand‐washing – is dependent on the type of water or sanitation facility. Table 1 lists types of improved and unimproved water, sanitation and hygiene facilities according to WHO/UNICEF JMP (2017).

**Table 1:** JMP classification of water, sanitation and hygiene facilities

	Drinking water	Sanitation	Hygiene
**Improved facilities**	Piped supplies: Tap water in the dwelling, yard, or plot Public standposts/pipes Non‐piped supplies: Boreholes / tubewells Protected wells and springs Rainwater Packaged water, including bottled water and sachet water Delivered water, including trucks and small carts Improved sources that require less than 30 minutes round‐trip to collect are defined as ‘basic water’. Improved sources requiring more than 30 minutes are defined ‘limited water’.	Networked sanitation: Flush and pour flush toilets connected to sewers On‐site sanitation: Flush or pour flush toilets connected to septic tanks or pits Pit latrines with slabs Composting toilets, including twin pit latrines and container‐based systems Shared facilities of the above types are defined as ‘limited sanitation’.	Fixed or mobile handwashing facilities with soap and water (defined as ‘basic hygiene’): Handwashing facility defined as a sink with tap water, buckets with taps, tippy‐taps, and jugs or basins designated for handwashing. Soap includes bar soap, liquid soap, powder detergent, and soapy water. Handwashing facilities without soap and water (e.g. ash, soil, sand or other handwashing agent) are defined as ‘limited hygiene’
**Unimproved facilities**	Non‐piped supplies: Unprotected wells and springs.	On‐site sanitation or shared facilities of the following types: Pit latrines without slabs Hanging latrines Bucket latrines	
**No facilities**	Surface water (e.g. drinking water directly from a river, pond, canal or stream)	Open defecation (disposal of human faeces in open spaces or with solid waste)	No handwashing facility on premises

Source: Based on WHO/UNICEF (2017).

### Conceptual framework of the EGM

**Figure 1:**
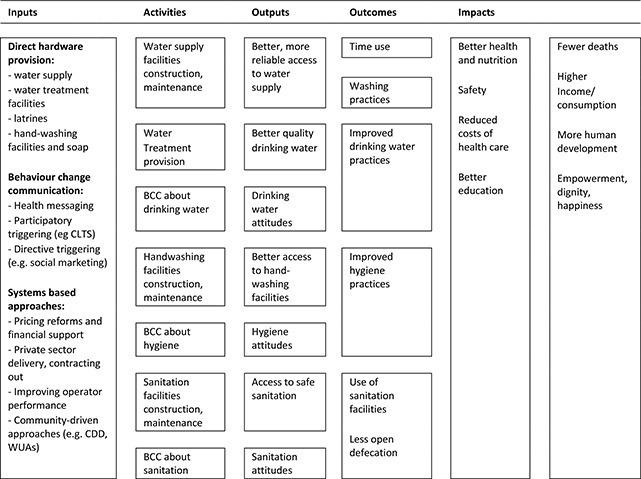
WASH interventions conceptual framework Source: authors based on White and Gunnarson (2008).

The conceptual framework links WASH interventions with impacts along the causal chain (Figure 1). Sector interventions – water and sanitation hardware and software provision and interventions in sector governance (e.g. contracting out and subsidies) – are presented to the left of the figure. Impacts on wellbeing – health, education, income and empowerment – are presented on the right. The conceptual framework shows the causal chain through which inputs are turned into final wellbeing impacts, through activities (construction of new facilities or behaviour change campaigns), outputs (better access to and quality of services) and outcomes (behaviour change, better use of those services).

The links in the chain are not automatic. For example, in the particular case of water quality, faecal contamination of drinking water between source and point‐of‐use (POU) means that hygienic approaches may be needed to store clean water collected at source, or treat water for contaminants in the household (POU). Better access to water supply (quantity) may improve health by reducing contamination in the environment by enabling better personal hygiene (e.g. handwashing) and environmental hygiene (e.g. safe disposal of faeces). Factors such as environmental faecal contamination may prevent impacts from clean drinking water provision being realised. Sustainability of impacts requires continued (permanent) adoption and acceptance by beneficiaries as well as appropriate solutions to reduce ‘slippage’ in improved behaviour and financial barriers to uptake and technical solutions to ensure service delivery reliability. Scalability requires that impacts which are demonstrated under ‘ideal settings’ of trials are achievable in the context of ‘real world’ programme implementation, where beneficiaries may not constantly be reminded to use technologies appropriately.

### Why it is important to develop the EGM?

Progress towards the Millennium Development Goals (MDGs) was uneven in the sector. The MDG drinking water target to “halve the number without access to safe drinking water (defined as access to water from an improved source within one kilometre of the household)” was declared met in 2012, but of those who did gain improved access to drinking water since 1990, supplies are mainly provided at the community level and are often unreliable (WHO/UNICEF, 2013). The MDG sanitation target to “halve the number without access to sanitation by 2015” was missed (UN, 2015).

The Sustainable Development Goals (SDGs) are aspirational, aiming for universal coverage by 2030, and adding targets for hygiene.^1^ The SDG targets are as follows (WHO/UNICEF JMP, 2017):

- To provide safe and affordable drinking water for all, measured by population using safely managed drinking water that is an improved drinking water source, located on premises, available when needed and free from contamination (SDG 6.1).
- To end open defaecation and provide adequate and equitable sanitation for all, measured by population using safely managed sanitation services and a basic handwashing facility with soap and water (SDG 6.2). Safely managed sanitation is defined as an improved facility where excreta is treated and disposed of in situ or off‐site.
- To ensure all men and women have access to basic services, including basic drinking water, sanitation and hygiene (SDG 1.4).

In order to move towards these ambitious targets, it is likely that substantial improvements in resource allocation will be needed to promote interventions which are effective in improving behaviours and outcomes in particular contexts. The purpose of this evidence gap map is to assist policy‐makers and practitioners in gaining access to evidence on the effectiveness of WASH interventions.

In 2014, 3ie produced an evidence gap map (EGM) on the effectiveness of WASH interventions in improving quality of life outcomes. That map includes evidence until February 2014 and only considered quality of life outcomes (health and non‐health) as primary outcomes. Behaviour change outcomes were included as secondary outcomes, provided the study also included primary outcomes. In addition, the map excluded interventions in health facilities. This update aims to capture studies conducted since, as well as broadening the included interventions and outcomes to better reflect the state of evidence on WASH in 2018.

### Existing evidence maps and relevant systematic reviews

In 2014, 3ie produced an evidence gap map for household and community interventions for promoting water, sanitation, and hygiene consumption in LMICs.^2^ The present study is an update of that map. We are updating the searches and the scope of that map to incorporate: 1) behaviour change as a primary outcome; and 2) water, sanitation and hygiene interventions based in health facilities to improve maternal and child health. A large number of impact evaluations and systematic reviews of WASH interventions will be incorporated in the map. For example, Table 2 lists some reviews of interventions for water, sanitation and hygiene promotion in households and communities, many published prior to 2014.

**Table 2:** Systematic reviews of WASH interventions

**Outcomes**	Systematic reviews
**Diarrhea**	Curtis & Cairncross 2003 Gundry et al. 2004 Fewtrell & Colford 2004 (also published as Fewtrell et al. 2005) Arnold and Colford 2007 Clasen et al. 2007 Ejemot et al. 2008 Waddington et al. 2009 Hunter 2009 Clasen et al. 2010 Cairncross et al. 2010 Norman et al. 2010
**Respiratory infections**	Aiello et al. 2008 Rabie and Curtis 2006
**Helminth infections**	Ziegelbauer et al. 2012
**Trachoma**	Ejere et al. 2012
**Arsenic contamination**	Jones‐Hughes et al. 2013
**Nutrition**	Dangour et al. 2013
**Education**	Jasper et al. 2012 Birdthistle et al. 2011
**Income**	Turley et al. 2013
**Attitudes and behaviour**	Null et al. 2012 De Buck et al. 2017

## Objectives

The overarching aim of the evidence map is to gather and present the rigorous empirical research on the effectiveness of interventions to improve consumption of water, sanitation and hygiene in the household, communities, schools and health facilities. This protocol provides the project plan for an update to the 2014 WASH evidence gap map (EGM) to take stock of the existing evidence, and capture newly published work, on the effects of interventions in these areas.

The aim of the EGM is to identify, map, and describe existing evidence on the effects of interventions to improve access to, and quality of, WASH infrastructure, services, and practices in low‐ and middle‐income countries. This update of the original map aims to capture additional studies conducted in the last three years and extend the scope of the EGM, in particular to cover behavioural outcomes and WASH interventions at healthcare facilities. The primary outcomes for this gap map include morbidities (e.g. diarrhoea), mortality, psychosocial health, nutritional status, education, income, and time use. In addition, behavioural outcomes will also be included as primary outcomes, such as water treatment practices, hygiene behaviour, and latrine construction in CLTS.

The update of the EGM addresses three objectives:

(1) To identify existing evidence from high quality impact evaluations and systematic reviews (SRs), particularly those published since 2014, which can be used to inform policy.
(2) To expand the scope of the EGM to better capture WASH behaviour change and programmes implemented at healthcare facilities, with the aim of improving the map's policy relevance.
(3) To identify existing gaps in evidence where new primary studies and systematic reviews could add value.

The results from this EGM aim to inform the direction of future research surrounding WASH, and discussions based on systematic evidence about which approaches and interventions are most effective in the WASH sector, whether they are used in small scale projects or large scaled‐up programmes.

## Methodology

### Defining evidence and gap maps

Evidence gap maps aim to establish what we know, and do not know, about the effectiveness of interventions in a thematic area (Snilstveit et al., 2016).^3^ The evidence gap map presented here includes evidence from primary studies and systematic reviews. It provides a graphical display of interventions and outcomes, indicating the density and paucity of available evidence, and gives confidence ratings for systematic reviews. Evidence gap maps articulate absolute gaps, which are filled with new primary studies, and synthesis gaps, which are filled with new systematic reviews and meta‐analyses. They are global public goods which attempt to democratise high quality research evidence for policy makers, practitioners, the public and research commissioners.

**Table 3:** Intervention mechanism classifications

**Mechanism of delivery**	Sub‐categories	Interventions
**Direct provision**	None	The provision of any WASH hardware for free and which has been chosen by an external authority. This includes interventions where soap is handed out, water purifiers given away, or latrines built by external actors.
**Health messaging**	None	Directive hygiene, and sometimes sanitation, education where participants are provided with new knowledge or skills to improve their health. These information campaigns may be provided by television, radio, or printed media; provided directly to specific households or through sessions at community meetings / schools / etc.; or provided directly to community leaders or health workers.
**Psychosocial ‘triggering’**	Directive	Psychosocial ‘triggering’ covers campaigns that use emotional and social cues, pressure, or motivation to encourage community members to change behaviours. Directive mechanisms are typically social marketing campaigns, which use commercial marketing techniques to promote the adoption of beneficial behaviours.
	Participatory	Participatory mechanisms are typically a community‐based approach and promote behaviour change through consultation with the community, a two‐way dialogue, and joint‐decision making. Community‐led total sanitation (CLTS) is the most common intervention with this mechanism.
**Systems‐based approaches**	Pricing reform	This covers all interventions that aim to change behaviour, such as the use of a technology, through changing the price of the requisite hardware. This includes subsidies and vouchers aimed at consumers.
	Improving operator performance	These interventions improve access to WASH facilities and services by improving the functioning of the current service provider. This includes improving accountability, increasing oversight/regulation, and changing the financing structure.
	Private sector (PS) and small‐scale independent providers (SSIPs) involvement	These interventions encourage the private sector, including not for profits, to become the providers of WASH facilities and services on a commercial basis.
	Community driven development (CDD)	CDD is a form of decentralised delivery that focuses on putting the community at the centre of the planning, design, implementation, and operations of their service provider. It typically uses a participatory approach, cost sharing, and often a component of local institutional strengthening. It includes social funds.
**Multiple mechanisms**	Direct provision with health messaging	These interventions combine the direct provision of hardware with an intensive health messaging campaign. If only a single session is provided to explain the new hardware, this would simply appear under “direct hardware provision”.
	Direct provision with psychosocial ‘triggering’	These interventions combine the direct provision of hardware with behavioural change communication that uses psychosocial triggers; these can be either participatory or more often directive (e.g. a social a marketing campaign).
	Systems‐based approaches with health messaging	These interventions combine systems‐based approaches (e.g. subsidies) with health messaging.
	Systems‐based approaches with psychosocial ‘triggering’	These interventions combine systems‐based approaches with behavioural change communication that uses psychosocial triggers.

### The framework

The framework for this evidence map (Appendix A) is based on the previous WASH evidence map framework developed by the authors (see footnote 2). However, the framework was updated based on a review of the academic and policy literature, and in consultation with relevant decision makers and other key stakeholders (see stakeholder engagement below). The included systematic reviews and impact evaluations will be identified through a comprehensive search of published and unpublished literature. It will include both completed and on‐going studies to help identify research in development that might help fill existing evidence gaps.

The finalised updated evidence map will be structured around a framework of policy relevant WASH mechanisms and outcomes, with a filter for technologies, and will be available online at 3ie's evidence gap map portal.^4^ Key features include:

**Table 4:** Intervention technology classifications

**WASH technologies**	Sub‐categories	Interventions
**Water Supply**	Source	New or improved water supply or distribution methods that do not provide the water directly to households. This includes boreholes or standpipes that require travel for water collection.
	Point of use (POU)	New or improved water supply or distribution methods that provide water directly to the household or at a communal point that requires no travel (i.e. in a garden shared by 20 houses). This includes water directly piped to houses or standpipes within the near vicinity.
**Water Quality**	Source	Supplies for, and information on, wither water treatments to remove microbial contaminants or safe water storage practices at a communal water access point.
	POU	Supplies for, and information on, water treatments to either remove microbial contaminants or safe water storage practices within the household or commune.
**Sanitation hardware**	Latrines	New or improved hardware for latrines or other means of excreta disposal.
	Sewer connection / drainage system	Connecting existing means of excreta disposal to a sewer or other drainage system.
**Hygiene**	Soap or hand sanitiser	Soap or similar products (e.g. hand sanitiser) with information on how to properly use them.
	Other hygiene supplies	Toilet paper, sanitary towels, or other hygiene products with information on how to correctly use them.
	Improved handwashing practices	Knowledge on the best practices for handwashing.
	Other improved hygiene practices	Knowledge on the best practices for other hygiene techniques or procedures (including face washing, menstrual hygiene, and latrine cleaning). This category also includes personal food hygiene practices beyond handwashing at appropriate times. This includes covering and storing food properly and washing dishes effectively.
**Multiple WASH**	Combined water supply and sanitation (WSS) programmes	Programmes that provide water supply and sanitation technologies.
	Other combinations	All other programmes that provide multiple technologies.

- The evidence map will highlight the best available evidence from systematic reviews and provide access to user‐friendly summaries and appraisals of those studies.
- The evidence map will also show where completed and, through the inclusion of trial registries, on‐going primary studies (impact evaluations) have been conducted.
- The evidence map will highlight absolute gaps in evidence (lack of studies for particular interventions and/or outcomes).
- The evidence map will highlight synthesis gaps where there are sufficient studies for a new systematic review or an update of an existing systematic review.

The evidence map will have filters to highlight the evidence in particular countries and regions, targeted at particular populations, using specific methodologies, and specific intervention approaches.

#### Population

We will include any study populations regardless of age, sex or socio‐economic status. However, populations are restricted to those in low‐ and middle‐income countries (LMICs), as defined by the World Bank, at the time the research was carried out.

#### Intervention

Water, sanitation, and hygiene can be categorised into groups and sub‐groups of related intervention mechanisms as shown in Table 3 (De Buck et al., 2017 and Poulos et al., 2006). We have aimed to define our mechanism categories so that all common WASH programmes would be eligible based on mechanism.

We then propose that the interventions should be further classified into groups and sub‐groups of related WASH technologies as shown in Table 4 (Esrey, 1991; Fewtrell et al., 2005; Waddington et al., 2009). We have aimed to define our technologies so that all common personal and household WASH interventions would be eligible based on technology.

As mentioned before, we will also be integrating an ecological model focused on where a technology is physically used. Ecological models stress the importance of the dynamic relationships between the personal and environmental factors that shape an individual's behaviours and lifelong human development. They, and their socio‐ecological counterparts, are often applied in crime prevention (for an example see Wortley, 2014) to explain why changes in city planning and the physical space of a neighbourhood can affect crime rates. Here we will separate out the hardware by place of use to emphasise the differential effect, and potentially different theories of change, of providing the same technology in different locations. The place of use affects both the convenience of the technology, and therefore why and how much it is adopted, as well as how it is expected to disrupt the transmission of disease. For example, providing a latrine to a household is a very different intervention to providing one at a school or a shared one to a community. The four main spaces in which WASH technologies for personal consumption are provided are within a home (for the use of a single household), within a shared community space (for example, a public water source such as a communal tubewell), at a school, and at a health facility,

There are different combinations and ways of presenting evidence from both multiple mechanisms and technologies, which we will consider further during the data extraction phase.

We will exclude all studies without a clearly defined WASH intervention. Programmes that combined a WASH intervention with a non‐WASH one will be included if the WASH component is defined as a primary, not secondary, element.

*Outcomes*

We will include studies that report the following types of quality of life outcomes:

(1) Health impacts including, but not necessarily limited to:
  a. diarrhoeal disease
  b. acute respiratory infections (ARIs)
  c. other water related infections such as helminths
  d. pain and musculoskeletal disorders
  e. psychosocial health and safety
  f. reproductive health outcomes
  g. mortality.
(2) Nutritional impacts including, but may not be limited to:
  a. measures of stunting (e.g. height‐for‐age Z‐scores, HAZ)
  b. wasting (e.g. weight‐for‐height Z‐scores, WHZ, and body mass index, BMI)
  c. underweight (e.g. weight‐for‐age Z‐scores, WAZ).
(3) Social and economic impacts, for example:
  a. educational outcomes (e.g. absenteeism)
  b. time use
  c. labour market outcomes (e.g. employment and wage)
  d. measures of income, consumption, and income poverty.

We expect most studies will focus on outcomes among children but would not exclude studies that only report outcomes for adults.

(4) We will also include studies even if they only report on the following types of behavioural and attitudinal outcomes:
  a. water quantity used/consumed
  b. water treatment practices
  c. latrine use or defaecation practices (including construction of facilities for ‘triggering’ interventions)
  d. hygienic behaviour (e.g. observed hand washing practices, measurement of hand contamination, microbial food contamination)
  e. willingness to pay.

We will exclude studies that only report measures of knowledge and attitudes; for example, a hygiene education programme that reports the proportion that know that bacteria can cause infections would be excluded.

Any adverse or unintended outcomes found to be reported, but not captured in the above list, will be included.

### Criteria for including and excluding studies

#### Types of study designs

This evidence gap map will include impact evaluations and systematic reviews of the effectiveness of technologies and intervention mechanisms. Impact evaluations are defined as programme evaluations or field experiments that use quantitative approaches applied to experimental or observational data to measure the effect of a programme relative to a counterfactual representing what would have happened to the same group in absence of the programme. Impact evaluations may also test different programme designs. We will include both completed and on‐going impact evaluations and systematic reviews; to capture the latter, we will include prospective study records in trial registries or protocols when available. We will include a broad range of intervention study designs in order for the map to provide a comprehensive look at the evidence provided by researchers working in the sector in different disciplines (e.g. epidemiology, econometrics). We include randomised and non‐randomised controlled studies. We also include methods such as natural experiments which may provide ‘as‐if randomised’ evidence when well conducted (Waddington et al., 2017). We allow broader inclusion criteria for particular outcomes, such as case‐control studies in the case of mortality, which may not be ethically collected in trials, and uncontrolled before versus after for time‐use outcomes, which are crucially important for water collectors and which arguably do not require controls (White and Gunnarson, 2008).

Study design criteria for includable intervention studies are as follows:

a) Prospective studies allocating the participants to the intervention using randomised or quasi‐randomised mechanisms at individual or cluster levels.
  a. Randomised control trial (RCT) with assignment at individual or cluster level (e.g. clustering at village, school, health facility)
  b. Quasi‐RCT using a quasi‐random method of prospective assignment (e.g. alternation of clusters)
b) Non‐randomised designs with selection on unobservables:
  a. Natural experiments using methods such as regression discontinuity (RD)
  b. Panel data or pseudo‐panels with analysis to account for time‐invariant unobservables (e.g. difference‐in‐difference, DID, or fixed‐ or random‐effects models)
  c. Cross‐sectional studies using multi‐stage or multivariate approaches to account for unobservables (e.g. instrumental variable, IV, or Heckman two‐step estimation approaches)
c) Non‐randomised designs with selection on observables:
  a. Cross‐sectional or panel (controlled before and after) studies with an intervention and comparison group using methods to match individuals and groups statistically (e.g. PSM) or control for observable confounding in adjusted regression.
d) The following impact evaluation study designs will only be included in the specific circumstances described.
  a. Reflexive controls (pre‐test/post‐test with no control/comparison group) will be included for studies reporting time use outcomes.
  b. Case‐control and cross‐sectional exposure designs will be included for studies conducted at healthcare facilities measuring mortality.
e) Studies explicitly described as systematic reviews and that describe methods used for search, data collection, and synthesis.

We will include impact evaluations where the comparison/control group receive no intervention (standard WASH access), a different WASH intervention, a placebo (e.g. school books) or the study employs a pipeline (wait‐list) approach.

#### Treatment of qualitative research

We do not plan to include qualitative research.

#### Types of settings

All included impact evaluations must have been conducted in low‐ and middle‐income countries (LMICs) as defined by the World Bank at the time of the intervention. We will also exclude systematic reviews only containing evidence from high‐income countries. We will include studies in challenging circumstances such as refugee camps, but exclude studies which are conducted under outbreak conditions, such as epidemics of cholera (‘extremely watery diarrhoea’) as this map aims to describe the evidence on what works under normal conditions.

As we are focusing on personal and household WASH interventions, we will exclude studies that look at WASH interventions in agriculture, commercial food preparation, and ones that focus on animal excreta. We will, however, include WASH interventions at medical facilities if they meet the above intervention definitions. Studies on medicalised hygiene (such as sterilising wounds) will be excluded.

#### Status of studies

We will search for and include completed and on‐going studies. We will not exclude any studies based on language or publication status or publication date.

### Search strategy and status of studies

We will automatically include all studies that were included in the 2014 evidence gap map for which thorough searches were conducted for both published and ‘grey’ literature. Therefore, the search strategy will cover two main components: updating the searches already conducted from February 2014 onwards, and conducting new electronic searches for the expanded scope from 2000 onwards. We will use the following strategies to identify completed and on‐going new potential studies:

1) Database and trial registry searches: We will search MEDLINE(R) (Ovid), Embase (Ovid), CAB Global Health (Ovid), CAB Abstracts (Ovid), Cochrane Library, ERIC (Proquest), Social Sciences Citation Index (Web of Science), Social Sciences Premium Collection (Proquest), Popline, WHO Global Health Library, Econlit (Ovid), Ebsco Discovery, and Campbell Library.
2) Organisation searches: We will search for literature using 3ie's impact evaluation database and through the online repositories of organisations who are known to produce impact evaluations and systematic reviews of WASH interventions. These include the Asian Development Bank, African Development Bank, Inter‐American Development Bank, Department for International Development, IMPROVE International, International Water and Sanitation Centre (IRC), Oxfam, UNICEF, US Agency for International Development, WaterAid, the World Bank (DIME, Impact Evaluations, IEG) (Table 5).
3) Bibliographic searches: Several recent systematic reviews (e.g. De Buck et al., 2017; Benova, et al., 2014) are relevant to topics in our expanded scope and we will screen these systematic reviews to locate additional primary studies.
4) We will also conduct bibliographic back‐referencing of reference lists of all included systematic reviews to identify additional primary studies and systematic reviews.

**Table 5:** Organisation hand‐searches

**Organizations**	Website
**3ie**	3ie water and sanitation sector
**Abdul Latif Jameel Poverty Action Lab (J‐PAL)**	J‐PAL evaluations
**African Development Bank**	African Development Bank evaluation reports
**Asian development Bank**	ADB Impact evaluation studies
**CEGA (University of California Center for Effective Global Action)**	CEGA water and sanitation research projects
**Department for International Development Research for Development**	Research for Development outputs in water and sanitation
**IMPROVE International**	WASH organisations with independent evaluations
**International Water and Sanitation Centre (IRC)**	http://www.washdoc.info/docsearch/results?lmt=20&txt=water+sanitation+impact+evaluation&combine=all&field=&language=&mediatype=&dateset=since&date=0
**Innovations for Poverty Action (IPA)**	IPA WASH projects
**Inter‐American Development Bank**	Office of Evaluation and Oversight: project and impact evaluations in water & sanitation
**Oxfam**	Water sanitation and hygiene evaluation and research reports
**USAID**	USAID development experience clearinghouse
**UNICEF**	UNICEF evaldatabase
**World Bank Development Impact Evaluation (DIME)**	DIME)
**World Bank Independent Evaluation Group (IEG)**	IEG Systematic reviews and impact evaluations

Appendix B presents an example of the search strings used for publication databases and search engines; it includes terms for the suitable interventions, regions, and methodologies.

### Screening and selection of studies

We will use EPPI reviewer to assess studies for inclusion at both the title / abstract and full‐text screening stages. Due to time and resource constraints, at the title / abstract stage, we will use EPPI reviewer's machine learning capabilities to prioritise studies in order of likelihood of inclusion. We will screen until we are no longer finding any studies to include (at least 50 studies with 0 includes). Two researchers will screen each title / abstract and each full‐text. Any disagreements on inclusion will be resolved through discussion.

### Data extraction, coding and management

For impact evaluations, we will use a standardised data extraction form to extract descriptive data from all studies meeting our inclusion criteria. Data extracted from each study will include bibliographic details, intervention types and descriptions, outcome types and descriptions, study design, context / geographical information, details on the comparison group, and on the quality of the implementation. We will also extract data on the sex disaggregation of outcomes.

A full list of data to be extracted is described in the coding tool in Appendix C; this tool will be piloted to ensure consistency in coding and resolve any issues or ambiguities. A single researcher will conduct the data extraction for each study; however, all coders will be trained on the tool before starting and a sample will be double coded to check for consistency.

For systematic reviews, a modified version of the tool will be developed for the data extraction. All systematic reviews for inclusion will undergo a critical appraisal following the 3ie systematic review database protocol for appraisal of systematic reviews (3ie, n.d.). Critical appraisals will be completed by one experienced researcher.

### Quality Appraisal

We will critically appraise included systematic reviews according to the 3ie tool (3ie, n.d.) which draws on Lewin et al. (2009). The tool appraises systematic review conduct, analysis and reporting, guiding appraisers towards an overall judgement of low, medium and high confidence in the review findings. We will assess primary studies on design only (randomised versus non‐randomised assignment and method of analysis).

We will not be critically appraising the quality of the included impact evaluations, but will collect data on study design. For the purpose of the present map it is not necessary to critically appraise the impact evaluations, beyond indicating whether the evidence is from randomised, natural experiment, or non‐randomised studies, as the systematic reviews provide overviews of the body of evidence, including their quality, where they exist. A major purpose the map is to provide access to the body of work on particular outcomes and interventions to encourage further syntheses of those studies by WASH sector researchers.

## Analysis and Presentation

### Unit of Analyses

Where multiple papers exist on the same study (e.g. a working paper and a published version), the most recent open access version will be included in the evidence map. If the versions report on different outcomes, an older version will be included for the outcomes not covered in later versions.

### Planned analyses

The matrix and filters are described above and in Appendix A. In brief, the matrix will display interventions mechanisms (direct provision, health messaging, psycho‐social triggering and systems‐based approaches) against outcomes along the causal chain (behaviour change and attitudes, health outcomes, nutrition outcomes, socio‐economic outcomes). It will be searchable by several filters including WASH technology (water quantity, water quality, sanitation, hygiene, multiple interventions), location (households, communities, schools and health facilities), study design (randomised and non‐randomised assignment), country and global region, and location (rural, urban, slum, refugee camp). The report will include descriptions of the evidence base according to these categories and present a global map, tables and figures presenting descriptive information about these characteristics. The report will present separately evidence from primary research (impact evaluations) and synthesis (systematic reviews).

### Presentation

The matrix and filters are described above and in Appendix A. In brief, the matrix will display interventions mechanisms (direct provision, health messaging, psycho‐social triggering and systems‐based approaches) against outcomes along the causal chain (behaviour change and attitudes, health outcomes, nutrition outcomes, socio‐economic outcomes). It will be searchable by several filters including WASH technology (water quantity, water quality, sanitation, hygiene, multiple interventions), location (households, communities, schools and health facilities), study design (randomised and non‐randomised assignment), country and global region, and location (rural, urban, slum, refugee camp).

## Stakeholder engagement

We have engaged stakeholders on the evidence matrix at various organisations who provide WaSH sector policy and programmes. These include Aga Khan Foundation, the Independent Evaluation Group of the World Bank, Japan International Cooperation Agency, Sanitation and Hygiene Applied Research for Equity (SHARE) consortium, the Water Supply and Sanitation Collaborative Council, and WaterAid.

## EGM authors

**Lead EGM author:** The lead author is the person who develops and co‐ordinates the EGM team, discusses and assigns roles for individual members of the team, liaises with the editorial base and takes responsibility for the on‐going updates of the EGM.

**Table:** 

Name:	Hugh Waddington
Title:	Senior Evaluation Specialist
Affiliation:	International Initiative for Impact Evaluation (3ie)
Address:	London International Development Centre 36 Gordon Square
City, State, Province or County:	London
Post code:	WC1H 0PD
Country:	UK
Phone:	+44 207 958 8350
Email:	hwaddington@3ieimpact.org
**Co‐authors:**	
Name:	Hannah Chirgwin
Title:	Research Associate
Affiliation:	3ie
Address:	London International Development Centre 36 Gordon Square
City, State, Province or County:	London
Post code:	WC1H 0PD
Country:	UK
Phone:	+44 207 958 8352
Email:	hchirgwin@3ieimpact.org
	
Name:	John Eyers
Title:	Information Retrieval Specialist
Affiliation:	3ie
Name:	Yashaswini PrasannaKumar
Title:	Public Policy Research Consultant
Affiliation:	3ie
Name:	Duae Zehra
Title:	Research Assistant
Affiliation:	University College London
Name:	Sandy Cairncross
Title:	Professor
Affiliation:	London School of Hygiene and Tropical Medicine

## Roles and responsibilities

- Content: Sandy Cairncross has substantial expertise in water, sanitation and hygiene interventions for the control of disease. Hugh Waddington led a previous review of WASH impacts.
- Systematic review methods: Sandy Cairncross has led several major systematic reviews of WASH interventions. Hugh Waddington led a previous systematic review and has supported a large number of systematic reviews and meta‐analyses for 3ie and Campbell.
- Information retrieval: John Eyers has substantial expertise in devising and running systematic searches for published and unpublished literature, including for reviews of WASH interventions.

## Sources of support

We thank JICA and the Water Supply and Sanitation Collaborative Council (WSSCC) for financial support. Ritsuko Yamagata, Jeff Tanner, Midori Makino, Ryotaro Hayashi and Marie Gaarder provided helpful comments.

## Declarations of interest

Sandy Cairncross has been involved in the development of sanitation and hygiene interventions and he and Hugh Waddington have authored primary studies and systematic reviews that may be eligible for inclusion in the evidence map. Inclusion decisions and critical appraisal of any studies these two authors have been involved in will be undertaken by other members of the team. We are not aware of any other conflicts that might affect decisions taken in the review and results presented.

## Preliminary timeframe

Approximate date for submission of the EGM: July 2018.

## Plans for updating the EGM

This map is itself an update of a EGM published online in 2014. We plan to update the map (or support others in doing so) when sufficient further studies and resources become available.

## Appendix

**Table:**

		**Behavioural Impacts**	**Health Impacts**	**Nutrition Impacts**	**Socio‐economic impacts**
		Water quantity consumed / used Water treatment practices Sanitation behaviour (incl. construction of facilities for triggering interventions) Hygiene behaviour Time use Willingness to Pay	Morbidity: diarrhoea Morbidity: acute respiratory infection (ARIs) Morbidity: other water related infections (e.g. helminths) Morbidity: other health outcomes Psychosocial health (e.g. happiness) Mortality	Nutrition / anthropometry	Education Labour market outcomes Safety Income / consumption / poverty
**Direct provision**					
**Health messaging**					
**Psychosocial ‘triggering’**	Directive				
Participatory					
**Systems‐based approaches**	Pricing reform Improving operator performance Private sector (PS) and small‐scale independent providers (SSIPs) involvement Community driven development (CDD)				
**Multiple mechanisms**

**Table:**

**Filter ‐ Type of technology**
Water supply: community provision
Water supply: household provision
Water supply: at school
Water supply at health facility
Water quality: community provision
Water quality: household provision
Water quality: at school
Water quality: at health facility
Sanitation: latrines for communal use
Sanitation: latrines for household use
Sanitation: latrines at school
Sanitation: latrines at health facility
Sanitation: safe waste disposal system (e.g. sewer connection/drainage system)
Hygiene: improved handwashing practices
Hygiene: handwashing supplies (e.g. soap or hand sanitiser) for household
Hygiene: handwashing supplies (e.g. soap or hand sanitiser) at school
Hygiene: handwashing supplies (e.g. soap or hand sanitiser) at health facility
Hygiene: menstrual care
Hygiene: other improved practices (incl. face washing)
Hygiene: combined technologies
Combined water supply and sanitation (WSS)
Combined water and hygiene
Combined sanitation and hygiene
Combined water, sanitation, and hygiene

## Appendix

Below are search strings for the searches formatted for Econlit on the Ovid search platform. These will be adapted for different databases and expanding the scope where necessary.

*Low‐ and middle‐income countries filter:*

1. (Africa or Asia or Caribbean or West Indies or South America or Latin America or Central America).ti,ab,hw.
2. (Afghanistan or Albania or Algeria or Angola or Argentina or Armenia or Armenian or Azerbaijan or Bangladesh or Benin or Byelarus or Byelorussion or Belarus or Belorussian or Belorussia or Belize or Bhutan or Bolivia or Bosnia or Herzegovina or Hercegovina or Botswana or Brazil or Bulgaria or Burkina Faso or Upper Volta or Burundi or Urundi or Cambodia or Khmer Republic or Kampuchea or Cameroon or Cameroons or Cameron or Camerons or Cape Verde or Central African Republic or Chad or China or Colombia or Comoros or Comoro Islands or Comores or Mayotte or Congo Zaire or Costa Rica or Cote d'Ivoire or Ivory Coast or Croatia or Cuba or Djibouti or French Somaliland or Dominica or Dominican Republic or East Timor or East Timur or Timor Leste or Ecuador or Egypt or United Arab Republic or El Salvador or Eritrea or Fiji or Gabon or Gabonese Republic or Gambia or Gaza or Georgia Republic or Georgian Republic or Ghana or Gold Coast or Grenada or Guatemala or Guinea or Guiana or Guyana or Haiti or Honduras or Hungary or India or Maldives or Indonesia or Iran or Iraq or Jamaica or Jordan or Kazakhstan or Kazakh or Kenya or Kiribati or Korea or Kosovo or Kyrgrzstan or Kirghizia or Kyrgyz Republic or Kirghiz or Kirgizstan or Lao PSR or Laos or Lebanon or Lesotho or Basutoland or Liberia or Libya or Macedonia or Madagascar or Malagasy Republic or Malaysia or Malaya or Malay or Sabah or Sarawak or Malawi or Mali or Marshall Islands or Mauritania or Mauritius or Agalega Islands or Mexico or Micronesia or Middle East or Moldova or Moldovia or Moldovian or Mongolia or Montenegro or Morocco or Ifni or Mozambique or Myanmar or Myanma or Burma or Namibia or Nepal or Netherlands Antilles or New Caledonia or Nicaragua or Niger or Nigeria or Pakistan or Palau or Palestine or Panama or Paraguay or Peru or Philippines or Philipines or Phillipines or Phillippines or Papau New Guinea or Romania or Rumania or Roumania or Rwanda or Ruanda or Saint Lucia or St Lucia or Saint Vincent or St Vincent or Grenadines or Samoa or Samoan Islands or Navigator Island or Navigator Islands or Sao Tome or Senegal or Serbia or Montenegro or Seychelles or Sierra Leone or Sri Lanka or Solomon Islands or Somalia or Sudan or Suriname or Surinam or Swaziland or South Africa or Syria or Tajikistan or Tadzhikistan or Tadjikistan or Tadzhik or Tanzania or Thailand or Togo or Togolese republic or Tonga or Tunisia or Turkey or Turkmenistan or Turkmen or Uganda or Ukraine or Uzbekistan or Uzbek or Vanuatu or New Hebrides or Venezuela or Vietnam or Viet Nam or West Bank or Yemen or Yugoslavia or Zambia or Zimbabwe).tw.
3. ((developing or less* developed or under developed or underdeveloped or middle income or low* income or underserved or under served or deprived or poor*) adj (countr* or nation? Or population? Or world or state*)).ti,ab.
4. ((developing or less* developed or under developed or underdeveloped or middle income or low* income) adj (economy or economies)).ti,ab.
5. (low* adj (gdp or gnp or gorss domestic or gross national)).tw.
6. (low adj3 middle adj3 countr*).tw.
7. (lmic or lmics or third world or lami countr*).tw.
8. Transitional countr*.tw.
9. Developing countries.hw.
  *Intervention terms:*
10. (Sanitation or sewage or sewerage or wastewater or water suppl* or water quality or water quantit* or water standard* or domestic water or drinking water or ((water adj3 access*) or hygiene)).ti,ab,sh.
11. (handwash* or soap* or (hand* adj3 (wash* or hygiene or clean*))).ti,ab,sh
  *Outcome terms:*
12. (ARIs or (respiratory adj (disease* or infection* or illness*)) or sinusitis or common cold* or otitis media or pharyngitis or influenza or flu or coryza or laryngitis or epiglottis or croup or pneumonia or bronchitis or bronchiolitis or pertussis or whooping cough).ti,ab,sh.
13. (diarrh* or dysenter* or gastroenteritis or cholera* or “waterborne infection*” or enterotoxin* or enteric or enteritis or “Escherichia coli*” or “e coli” or rotavirus* or mortality or death*).ti,ab,sh.
14. ((time adj3 (saving* or allocate* or conum* or feth* or travel*) or (collect* adj3 (time* or behavio* or chore* or errand* or drudgery or burden* or inconvenien*))).ti,ab,sh.
15. ((back* adj3 (pain* or injur*)) or backpain or ((neck or spine or spinal0 adj3 (pain or injur*)) or neckpain or musculoskeletal or (joint adj3 (pain or disease* or injur*)) or arthriti* or osteoarthriti* or menstraut* or menses or menstrual or amenorrh* or dysmenorrh* or oligomenorrh*).ti,ab,sh.
  *Methods filter:*
16. (“quasi experiment*” or quasi‐experiment* or “random* control* trial*” or “random* trial*” or RCT or (random* adj3 allocat*) or matching or “propensity score” or PSM or “regression discontinuity” or “discontinuity design” or RDD or “difference in difference*” or difference‐in‐difference* or “diff in diff” or DID or “case control” or cohort or “propensity weighted” or propensity‐weighted or “interrupted time series” or (before adj5 after) or (pre adj5 post) or ((pretest or pre test) and (posttest or post test)) or “research synthesis” or “scoping review” or “rapid evidence assessment” or “systematic literature review” or “systematic review” or “meta‐analy*” or metaanaly* or “meta analy*” or “control* evaluation” or “control treatment” or “instrumental variable” or heckman or IV or ((quantitative or “comparison group*” or counterfactual or “counter factual” or counter‐factual or experiment*) adj3 (design or study or analysis)) or QED).ti,ab,kw.

## Appendix

**Table:**

**Number**	**ID**	**Description**	**Question**	**Coding**
Publication details				
1.01	ID	Unique study identifier	Surname of first author and year of publication	Author##
1.02	AUTHOR	First author	Surname, Initial.	Surname, Initial.
1.03	TITLE	Full title	Full title of paper	Open answer
1.04	SHORT_TITLE	Short title	Short version identifying paper	Open answer
1.05	DATE	Publication date	Year	YYYY
1.06	COUNTRY			Open answer
1.07	LOCATION			Open answer
1.08	REGION	Region of study	Which region was the study conducted in?	1=East Asia & Pacific (EAP) 2=Latin America & Caribbean (LAC) 3=Middle East & North Africa (MENA) 4=South Asia (SA) 5=Sub‐Saharan Africa (SSA)
*2. Intervention details*				
2.01	INTERVENTION_TECHNOLOGY	WASH technology received by participants	Indicate type of WASH technology received	1.1=Water supply: community provision 1.2=Water supply: household provision 1.3=Water supply: at school 1.4=Water supply at health facility 2.1=Water quality: community provision 2.2=Water quality: household provision 2.3=Water quality: at school 2.4=Water quality: at health facility 3.1=Sanitation: latrines for communal use 3.2=Sanitation: latrines for household use 3.3=Sanitation: latrines at school 3.4= Sanitation: latrines at health facility 3.5=Sanitation: safe waste disposal system (e.g. sewer connection/drainage system) 4.1=Hygiene: improved handwashing practices 4.2=Hygiene: handwashing supplies (e.g. soap or hand sanitiser) for household 4.3=Hygiene: handwashing supplies (e.g. soap or hand sanitiser) at school 4.4=Hygiene: handwashing supplies (e.g. soap or hand sanitiser) at health facility 4.5=Hygiene: menstrual care 4.6=Hygiene: other improved practices (incl. face washing) 4.7=Hygiene: combined technologies 5.1=Combined water supply and sanitation (WSS) 5.2=Combined water and hygiene 5.3=Combined sanitation and hygiene 5.4=Combined water, sanitation, and hygiene
2.02	MULTIPLE_TECHNOLOGY	Multiple technologies	Indicate complete list of all WASH technologies	1.1=Water supply: community provision 1.2=Water supply: household provision 1.3=Water supply: at school 1.4=Water supply at health facility 2.1=Water quality: community provision 2.2=Water quality: household provision 2.3=Water quality: at school 2.4=Water quality: at health facility 3.1=Sanitation: latrines for communal use 3.2=Sanitation: latrines for household use 3.3=Sanitation: latrines at school 3.4= Sanitation: latrines at health facility 3.5=Sanitation: safe waste disposal system (e.g. sewer connection/drainage system) 4.1=Hygiene: improved handwashing practices 4.2=Hygiene: handwashing supplies (e.g. soap or hand sanitiser) for household/community 4.3=Hygiene: handwashing supplies (e.g. soap or hand sanitiser) at school 4.4=Hygiene: handwashing supplies (e.g. soap or hand sanitiser) at health facility 4.5=Hygiene: menstrual care 4.6=Hygiene: other improved practices (incl. face washing) Indicate NA if 2.03 is not marked 4.7 ‐ 5.4
2.03	INTERVENTION_MECHANISM	Mechanism used to encourage/give WASH technology to participants	Indicate the mechanism by which the above technology was supplied to the individual/household/facility/community	1=Direct provision 2.1=Health messaging (incl. hygiene education): mass media 2.2=Health messaging (incl. hygiene education): household/community level 2.3=Health messaging (incl. hygiene education): training of trainers (incl. teachers, community leaders, medics) 2.4=Health messaging (incl. hygiene education): combined approach 3.1=Psychosocial ‘triggering’: directive (incl. social marketing) 3.2=Psychosocial ‘triggering: participative (incl. CLTS) 4.1=Systems‐based: pricing reform (incl. subsidies and vouchers) 4.2=Systems‐based: improving operator performance (incl. institutional reform, capacity building, operator financing, regulation, and accountability) 4.3=Systems‐based: private sector (PS) and small‐scale independent providers (SSIPs) involvement (incl. contracting out) 4.4=Systems‐based: community driven development (CDD, incl. social funds and community driven development) 5=Multiple mechanisms
2.04	MULIPLE_MECHANISM	Multiple mechanisms	Indicate all mechanisms used to deliver the technologies and write a brief description of what mechanism is used for what technology	1=Direct provision 2.1=Health messaging (incl. hygiene education): mass media 2.2=Health messaging (incl. hygiene education): household/community level 2.3=Health messaging (incl. hygiene education): training of trainers (incl. teachers, community leaders, medics) 3.1=Psychosocial ‘triggering’: directive (incl. social marketing) 3.2=Psychosocial ‘triggering: participative (incl. CLTS) 4.1Systems‐based: pricing reform (incl. subsidies and vouchers) 4.2=Systems‐based: improving operator performance (incl. institutional reform, capacity building, operator financing, regulation, and accountability) 4.3=Systems‐based: private sector (PS) and small‐scale independent providers (SSIPs) involvement (incl. contracting out) 4.4=Systems‐based: community driven development (CDD, incl. social funds and community driven development) Underneath the codes include an open answer description of what mechanism is used for what technology. Indicate NA if 2.05 is not marked 5
2.05	RURAL	Rural setting	Is study conducted in a rural area?	1=Yes 2=No
2.06	URBAN	Urban setting	Is study conducted in an urban or peri‐urban (end of urban) area?	1=Yes 2=No
2.07	SLUM	Slum setting	Is study conducted in a slum (informal settlement)?	1=Yes 2=No
2.08	REFUGEE	Refugee camp setting	Is study conducted in a refugee camp?	1=Yes 2=No
2.09	TARGET_GROUP	Intervention targeting	Which population groups did the intervention target?	Open answer (e.g. women, children, elderly, disabled)
*3. Study design and data collection*				
3.01	STUDY_DESIGN	Design type	Categorise the study design	1.1= Individually randomised controlled trial (RCT) 1.2= Cluster‐RCT 2.1= Quasi‐RCT (e.g. alternation) 2.2= Cluster‐quasi‐RCT 3= RDD (quasi‐experiment with discontinuity assignment) 4= CBA (quasi‐experiment with baseline and endline data collection in intervention and comparison group) 5= Cross‐section study (quasi‐experiment with endline data collection only in intervention and comparison group) 6= Reflexive control (before vs. after with no comparison group) 7= Cohort study 8= Case control study
3.02	STUDY_METHODS	Methods of inference	What methods are used to identify the treatment effect?	1=DID (difference‐in‐differences or fixed effects analysis) 2=PSM (statistical matching e.g. propensity‐score matching, coarse‐exact matching, propensity‐weighted regression) 3=Instrumental variables (IV)/Heckman selection (including switching regression) models 4=Multivariate regression/covariates‐adjusted analysis (e.g. ANCOVA analysis) 5=Bivariate analysis / comparison of means
*4. Impacts*				
4.01	IMPACT_VARIABLE	Impact or final outcome	What impacts are reported (code multiple outcomes in additional columns)?	1.1=Morbidity: Diarrhoea 1.2=Morbidity: Accute Respiratory Infections (ARIs) 1.3=Morbidity: Other water related infections (e.g. helminth infections) 1.4=Morbidity: Other health outcomes (e.g. drudgery, pain, musculoskeletal disorders) 1.5=Psycho‐social health (eg happiness) 1.6=Mortality 2.1=Nutrition/anthropometry (eg HAZ, WAZ, BMI) 3.1=Education (eg absenteeism) 3.2=Labour market outcomes (eg employment) 3.3=Safety 3.4=Income/consumption/poverty 4.1=Water quantity consumed/used (water supply behaviour change) 4.2=Water treatment practices (water quality behaviour change) 4.3=Sanitation behaviour (incl. construction of facilities for triggering interventions) 4.4=Hygiene behaviour 4.5=Time use 4.6=Willingness to pay 5=Cos‐benefit analysis
